# Changes in food-related costs during the COVID-19 pandemic among families managing food allergy

**DOI:** 10.3389/falgy.2022.915014

**Published:** 2022-07-15

**Authors:** Michael A. Golding, Cathérine Lemoine-Courcelles, Elissa M. Abrams, Moshe Ben-Shoshan, Philippe Bégin, Edmond S. Chan, Derek K. Chu, Jennifer D. Gerdts, Beatrice Povolo, Harold Kim, Elinor Simons, Julia Upton, Jennifer L. P. Protudjer

**Affiliations:** ^1^The Children's Hospital Research Institute of Manitoba, Winnipeg, MB, Canada; ^2^Department of Pediatrics and Child Health, The University of Manitoba, Winnipeg, MB, Canada; ^3^Division of Allergy and Immunology, Department of Pediatrics, University of British Columbia, Vancouver, BC, Canada; ^4^Division of Pediatric Allergy and Clinical Immunology, Department of Pediatrics, McGill University Health Center, Montreal, QC, Canada; ^5^Division of Clinical Immunology, Rheumatology, and Allergy, Department of Pediatrics, Sainte-Justine University Hospital Center, Montreal, QC, Canada; ^6^BC Children's Hospital Research Institute, Vancouver, BC, Canada; ^7^Department of Health Research Methods, Evidence and Impact, McMaster University, Hamilton, ON, Canada; ^8^Department of Medicine, McMaster University, Hamilton, ON, Canada; ^9^The Research Institute of St. Joe's Hamilton, Hamilton, ON, Canada; ^10^Food Allergy Canada, Toronto, ON, Canada; ^11^Department of Medicine, Western University, London, ON, Canada; ^12^Section of Allergy and Immunology, Department of Pediatrics and Child Health, University of Manitoba, Winnipeg, MB, Canada; ^13^Division of Immunology and Allergy, Department of Pediatrics, Hospital for Sick Children, University of Toronto, Toronto, ON, Canada; ^14^George and Fay Yee Center for Healthcare Innovation, Winnipeg, MB, Canada; ^15^Centre for Allergy Research, Karolinska Institutet, Stockholm, Sweden; ^16^Department of Food and Human Nutritional Sciences, The University of Manitoba, Winnipeg, MB, Canada

**Keywords:** food allergy, cost of illness, COVID-19, food-related costs, socio-economic status pandemic-related changes in allergy-friendly food purchasing 2

## Abstract

**Background:**

The COVID-19 pandemic has affected the supply, cost, and demand for certain foods, but it is not clear how these changes have affected food-allergic households.

**Objective:**

To describe the changes in food-related costs that have followed COVID-19, as reported by higher- and lower-income households with a food-allergic member.

**Methods:**

Between May 1-June 30, 2020, Canadian households, with at least one food-allergic member, completed an online survey on food shopping and preparation habits before and during the COVID-19 pandemic. The sample was divided into binary groups, either higher or lower than the sample median income. Data were analyzed using descriptive statistics and multiple regression.

**Results:**

The sample was comprised of 102 participants (i.e., 51/ income group). The three most common food allergies amongst both groups were peanuts, tree nuts and milk. Since the start of the pandemic, both groups reported greater monthly direct grocery costs, although costs amongst the higher-income group were twice as high as the lower-income group ($212.86 vs. $98.89, respectively). Indirect food preparation costs were similarly elevated. Higher-income households with food procurement difficulties reported increased indirect shopping costs following the outbreak of COVID-19, whereas those without such difficulties reported decreased costs. Lower-income households with allergies to milk, wheat, or eggs (i.e., staple allergy) experienced a larger change in indirect food preparation costs following the outbreak of COVID-19 relative to those with other food allergies ($244.58 vs. –$20.28, respectively; *p* = 0.03).

**Conclusion:**

Both higher and lower income households with food allergy reported greater direct food costs and indirect food preparation costs following the COVID-19. Households with staple allergy and those with difficulties finding their typical food items were particularly affected.

## Introduction

For the estimated 7% of families whose children live with food allergy (1), avoidance of the food to which they are allergic is essential to prevent a potentially fatal reaction (2). Owing to the perpetual need for dietary vigilance, children with food allergy and their families report worse health-related quality of life (3, 4) and mental health (5–7) than families not managing this condition. Prior to the COVID-19 pandemic, such vigilance also had substantial costs, including direct costs, such as the excess cost of food, estimated at, $2,500 more per year, on average, compared to families without food allergy, as well as indirect costs, attributable to time and opportunity costs (8).

The COVID-19 pandemic has caused significant changes in how individuals acquire, prepare and consume food. During the early months of the pandemic, supply chains, weakened by travel restrictions, physical distancing requirements, and workplace outbreaks, struggled to keep up with shifting dietary patterns. As a result, consumers reported difficulties finding many grocery items during the initial Spring 2020 wave of COVID-19 (9–11). Since that time, acute food shortages have become less of a concern, but consumers continue to be impacted by higher food prices (12–14). Although reductions in the availability of food and increased food prices likely affect all families, households with a food-allergic member(s) may bare a disproportionate burden given the restricted nature of their diet (15). Moreover, it is possible that the food-related changes that have followed COVID-19 may impact higher and lower income families differently, given their unique needs and financial resources. As such, the current study aimed to describe the changes in food-related costs that have followed COVID-19, as reported by higher and lower income households with a food-allergic member. We also investigated whether certain food allergy or household-level characteristics were predictive of cost changes. We hypothesized that food allergy-related household costs in the early months of the pandemic were higher than those prior to the pandemic.

## Methods

Participants were recruited from May 1st to June 30th 2020 through Food Allergy Canada, the national patient organization for those living with food allergy, and through social media. Canadians over the age of 18 years who self-reported a physician-diagnosed food allergy or who were living with someone with a physician-diagnosed food allergy were eligible to participate. Participants were excluded if they had an unclear diagnosis of food allergy, or reported any other chronic disease including, but not limited to celiac disease, diabetes or malignancies. The survey was posted online on food allergy social media pages, and was promoted by Food Allergy Canada, the largest food allergy patient organization in Canada. Participants were asked to complete an online, modified version of the Food Allergy Economic Questionnaire (FA-EcoQ), a validated, self-report measure designed to assess the direct (i.e., out of pocket) and indirect (i.e., loss of time or wages) costs of food allergy (16). Based on the information collected *via* the FA-EcoQ, the following cost variables (all reported in CAD) were derived:

*Change in direct grocery costs:* Monthly out-of-pocket grocery expenses during COVID-19 minus monthly out-of-pocket grocery costs prior to the pandemic.

*Change in indirect costs:* Described in detail elsewhere, (8) indirect costs were calculated by multiplying the number of hours devoted to food preparation and food shopping by the average hourly wage for non-unionized employees in Canada in the year 2020 ($26.27) (17). The time of unemployed respondents was valued at the average hourly minimum wage in Canada ($13.07). Indirect costs were calculated for the period prior to the pandemic and subtracted from pandemic costs to yield a change in food preparation costs variable and a change in food shopping costs variable.

Each of the cost variables were found to have missing data (amounts ranged from 3–16%); therefore, multiple imputation was used to create and analyze 70 datasets. Following imputation, participants were stratified into higher and lower income groups based on whether their monthly income fell above or below the sample median ($5,830 CAD) in order to better understand cost changes across income groups. The sample median was used for stratification not only because the income distribution was positively skewed, but also because it was comparable to the Canadian median ($4,642) (18) and provided two equal income groups. Means ± SDs were used to describe changes in food-related costs during COVID-19. Linear regression was used to identify factors associated with changes in food-related costs after adjusting for covariates. Data were analyzed using Stata Version 16.0 (College Station, TX). All participants provided their informed consent (University of Manitoba Health Research Ethics Board: HS22066).

## Results

### Participant demographics

In total, 102 patients participants were recruited, corresponding to 51 per income group (Table 1). In both groups, participants were predominantly female (>90%), approximately half reported 3–4 household members, and most managed one food allergy (higher income: 74%; lower income: 86%). The three most common food allergies amongst both groups were peanuts, tree nuts and milk. With consideration to EAI prescription, 90% of higher income families reported a prescription, compared to 71% of lower income families.

**Table 1:** Participant characteristics.

	**Participants above the sample median income (*****n*** = **51)**		**Participants below the sample median income (*****n*** = **51)**	
	**Frequencies**	**Mean (SD)**	**Frequencies**	**Mean (SD)**
Respondent age		45.7 (12.90)		44.52 (12.19)
Respondent sex				
Female	92%		94%	
Male	8%		4%	
Prefer not to say	–		2%	
Number of household members		3.43 (1.34)		3.04 (1.38)
1–2	25%		39%	
3–4	51%		45%	
4+	24%		16%	
Number of household allergies		1.29 (0.54)		1.18 (0.48)
1	74%		86%	
2	22%		10%	
3	4%		4%	
Common food allergies				
Peanut	71%		49%	
Tree nut	61%		51%	
Milk	29%		39%	
Egg	24%		22%	
Shellfish	31%		27%	
EAI prescription in the household				
Yes	90%		71%	
No	10%		29%	

### Cost comparisons between income groups

In the early months of the COVID-19 pandemic, both higher income and lower income groups reported that they spent more directly on groceries ($212.86 ±335.53 and $98.89 ± 285.56, respectively, per month) and indirectly on food preparation ($272.60 ± 360.36 and $130.23 ± 291.11, respectively) following the outbreak of COVID-19. In contrast, participants in both income groups reported decreased indirect shopping costs (–$14.80 ± 149.23 and; –$19.07 ± 142.44, respectively, per month), on average, over the same period.

### Predictors of monthly direct costs

Despite reporting higher total direct costs of $212 per month, on average, neither the type, number of household food allergies, severity of food allergy, nor the unavailability of typical foods, were predictive of greater costs amongst the higher income group (Table 2). A similar pattern was identified amongst lower the income group, except for the unavailability of typical foods, which was associated with, excess costs of $164.30 per month, on average (b=196.25; 95%CI 22.33 – 370.16). By comparison, participants in the lower income group with access to their typical foods reported spending less on food following the outbreak of the pandemic (i.e., -$31.94).

**Table 2:** Predictors of monthly direct grocery costs among higher and lower income households with food allergy.

	**Participants above the sample median income (*****n*** = **51)**				**Participants below the sample median income (*****n*** = **51)**			
	***b***	**SE**	**95% CI**	***p*–value**	***b***	**SE**	**95% CI**	***p*–value**
Monthly direct costs								
Staple allergy^†^	−19.95	117.51	−257.29; 217.39	0.87	−12.28	87.54	−189.81; 192.22	0.89
Severity^‡^	−101.25	109.36	−322.10; 119.60	0.36	−50.03	84.44	−220.96; 120.90	0.56
Number of household food allergies	−74.35	111.57	−299.68; 150.99	0.51	1.20	94.22	−189.81; 192.22	0.99
Typical foods unavailable	70.10	108.66	−149.35; 289.55	0.52	196.25	86.05	22.33; 370.16	0.03

*All analyses adjusted for number of household members, pandemic duration, and the provincial prevalence of COVID−19*.

† *Refers to households with a reported allergy to milk, wheat, or eggs*.

‡ *Severity is a binary variable that indexes whether a family member has experienced respiratory symptoms or loss of consciousness when exposed to food allergens*.

### Predictors of monthly indirect costs

Both income groups reported decreased indirect shopping costs, but increased food preparation costs in the early months of the pandemic. Amongst each income group, consideration to different types of indirect costs yielded differences (Table 3). With consideration to monthly indirect food preparation costs, no significant differences were found for higher income families, whereas lower income families with an allergy to milk, wheat, or eggs (i.e., staple allergy) experienced a larger change in indirect food preparation costs relative to those with other food allergies ($244.58 vs. –$20.28, respectively; b = 224.30; 95%CI 43.60–405.01, p = 0.03). Lower income families with severe disease reported a smaller change in indirect preparation costs relative those with less severe disease, albeit it did not reach statistical significance ($48.63 vs. $215.09, b = −166.46; 95%CI −334.53 – 1.61; p = 0.06). With consideration to monthly indirect shopping costs, higher income families who had difficulties finding their typical grocery items reported an increase in indirect shopping costs while those without such difficulties reported spending less time shopping ($34.09 vs. –$69.80, respectively; b = 103.89; 95CI 5.47–202.31). No such differences were identified amongst the lower income group.

**Table 3:** Predictors of monthly indirect food preparation and shopping costs among higher and lower income households with food allergy.

	**Participants above the sample median (*****n*** = **51)**				**Participants below the sample median (*****n*** = **51)**			
	***b***	**SE**	**95% CI**	***p*–value**	***b***	**SE**	**95% CI**	***p*–value**
Monthly indirect food preparation costs								
Staple allergy^†^	160.63	123.31	−89.03; 410.28	0.20	224.30	88.76	43.60; 405.01	0.02
Severity^‡^	−100.31	117.11	−337.06; 136.44	0.40	−166.46	82.92	−334.53; 1.61	0.06
Number of household food allergies	−201.85	117.20	−438.53; 34.83	0.09	−26.31	95.78	−221.30; 168.67	0.78
Typical foods unavailable	9.66	114.47	−221.52; 240.84	0.93	−85.38	91.10	−270.80; 100.04	0.36
Monthly indirect shopping costs								
Staple allergy	−7.79	51.55	−112.26; 96.67	0.88	32.11	47.55	−64.55; 128.78	0.50
Severity	−29.98	50.61	−132.95; 72.98	0.56	6.68	46.11	−86.93; 100.29	0.89
Number of household food allergies	27.50	51.20	−76.62; 131.63	0.60	−45.56	51.61	−150.53; 59.40	0.38
Typical foods unavailable	103.89	48.51	5.47; 202.31	0.04	−42.13	49.40	−142.64; 58.38	0.40

*All analyses adjusted for number of household members, pandemic duration, and the provincial prevalence of COVID-19*.

† *Refers to households with a reported allergy to milk, wheat, or eggs*.

‡ *Severity is a binary variable that indexes whether a family member has experienced respiratory symptoms or loss of consciousness when exposed to food allergens*.

## Discussion

During the COVID-19 pandemic, families managing food allergy reported higher direct and indirect food costs. However, important differences were noted by income strata. Higher income households reported increases in direct grocery costs twice as high as lower income households. Moreover, higher income households with difficulty procuring their typical foods reported increased indirect shopping costs, whereas trouble obtaining one's typical foods was associated with increased direct food costs for those in the lower income strata. Lastly, lower income households with allergies to staple foods reported increased food preparation costs.

At the time data were collected (between May-June 2020), grocery store visits across Canada were largely limited to one visit or less per week, and while food access was not reported to be problematic amongst the general population, there was a notable lack of availability of canned or frozen fruits and vegetables, and grain products (19). One possible explanation for the increased direct grocery costs among lower income food-allergic families with food procurement difficulties is that these families are obliged to buy more expensive alternatives when faced with product shortages. Unfortunately, for lower income families, the need to purchase more expensive products may cause financial strain, particularly in the current climate of financial uncertainty. In contrast, higher income food-allergic families appear not to experience a significant increase in direct costs in response to product shortages as it is likely that their typical items are already higher-priced. These greater increases in costs amongst higher income families may also reflect differences in baseline (pre-COVID) costs of food, a phenomenon that we previously reported (8).

Higher income families with food procurement difficulties did report greater *indirect shopping costs* following the outbreak of COVID-19, while lower income families did not. While it is difficult to know what accounts for this discrepancy, it is possible that it reflects the fact that lower wage workers are less likely to be afforded the opportunity to work remotely (20). Consequently, they may be reluctant to devote more time to shopping due to a lack of free-time or a desire to limit their exposure to COVID-19.

Findings from the current study also revealed a greater COVID-19 change in indirect food preparation costs among lower income families with an allergy to milk, wheat, or eggs. Given the ubiquity of these ingredients, it stands to reason that families with staple allergies would be more likely to rely on pre-packaged allergen-friendly products to meet their dietary needs. However, faced with higher grocery prices, lower income families with staple allergies may have shifted their consumption away from these convenient, but often expensive products, toward those requiring more preparation.

While the current study provides preliminary evidence of how the pandemic has affected the food-related costs of higher and lower income food-allergic households, its retrospective nature means that the pre-pandemic cost estimates could have been affected by recall bias. As participants were recruited online, confirmatory allergy testing was not possible. However, online recruitment was initiated on food allergy social media pages and email lists, and thus likely reached the intended audiences. The modest sample size means the study was, unfortunately, underpowered to detect small effect sizes. Given the small sample size and the exploratory nature of the research we decided not to control for family-wise error. However, failing to control for family-wise error does increase the risk of type 1 error. In light of these limitations, future research should utilize more objective measures of food costs and larger samples in order to better understand how the COVID-19 pandemic has affected households with food allergy. Such research is important to ensure that households with food allergy are not unduly affected by future supply chain disruptions, nor unexpected changes in demand. Prior to the COVID-19 pandemic, Canadian households managing food allergy were burdened with excess costs of approximately $200 per month, compared to households not managing food allergy (8). Owing to projected increases in food prices in 2022 (21), a continued focus on the impact of increasing food prices amongst those with medical dietary restrictions, such as food allergy, is warranted. Referrals to food allergy dietitians may be appropriate for families with the financial ability, either through household finances or insurance, to absorb these fees. Additional information, such as food substitutions and low-cost recipes, ought to be offered to families at the time of diagnosis. Finally, allergists are encouraged to be familiar with any food banks in their area that support families with allergy. While the prevalence of food insecurity amongst Canadian families generally has increased since the start of the COVID-19 (22), with the highest prevalence (19–22%) noted amongst households with children (22), families with food allergy are likely to be disproportionately affected, owing to higher food costs and fewer products from which to choose owing to medical dietary restrictions.

In conclusion, both higher and lower income households with food allergy reported greater direct food costs and indirect food preparation costs following the outbreak of COVID-19. Households with staple allergy and those with difficulties finding their typical food items were particularly affected.

## Data availability statement

The original contributions presented in the study are included in the article/supplementary material, further inquiries can be directed to the corresponding author/s.

## Ethics statement

The studies involving human participants were reviewed and approved by University of Manitoba Health Research Ethics Board: HS22066. The patients/participants provided their written informed consent to participate in this study.

## Author contributions

MG performed the statistical analysis and wrote the first draft of the manuscript. CL-C led the French translation of the survey instrument and contributed to the study design. EA, JG, and ES contributed to the study design, provided constructive comments on the manuscript, and including interpretation of the data. MB-S, PB, EC, DC, BP, HK, and JU provided constructive comments on the manuscript and including interpretation of the data. JP led the study design, secured funding for this work, contributed to the statistical analysis, and writing of the manuscript. All authors contributed to the article and approved the submitted version.

## Funding

Funding for this project was provided by the Canadian Institutes of Health Research through their Early Career Investigator in Maternal, Reproductive, Child and Youth Health Operating Grant, awarded to JP. JP's New Investigator Grant, jointly provided by the Manitoba Medical Services Foundation and the Children's Hospital Research Institute of Manitoba, also helped to support the current study. These funding bodies had no influence on the study design, data collection, analysis and interpretation, or manuscript writing.

## Conflict of interest

PB reports personal fees from Novartis, Pfizer, Sanofi, ALK and Aralez, as well as grants from DBV technologies, Regeneron and Sanofi outside the submitted work. EC has received research support from DBV Technologies and has been a member of advisory boards for Pfizer, Pediapharm, Leo Pharma, Kaleo, DBV, AllerGenis, Sanofi Genzyme, Bausch Health, and Avir Pharma. HK has served on the following speakers' bureau and/or advisory boards: AstraZeneca, Aralez, Bausch Health, CSL Behring, GSK, Kaleo, Novartis, Pediapharm, Pfizer, Sanofi, Shire, and Takeda. JU reports research support/grants from Novartis, Regeneron, ALK, DBV, CIHR, and fees from AstraZeneca, Pfizer, ALK Abello, Bausch Health, Kaleo, Food Allergy Canada, all outside the submitted work. JP sits on the steering committee for Canada's National Food Allergy Action Plan and is Section Head, Allied Health, Canadian Society of Allergy and Clinical Immunology. The remaining authors declare that the research was conducted in the absence of any commercial or financial relationships that could be construed as a potential conflict of interest.

## Publisher's note

All claims expressed in this article are solely those of the authors and do not necessarily represent those of their affiliated organizations, or those of the publisher, the editors and the reviewers. Any product that may be evaluated in this article, or claim that may be made by its manufacturer, is not guaranteed or endorsed by the publisher.
